# High-Performance Red Transparent Quantum Dot Light-Emitting Diodes via Fully Solution-Processed MXene/Ag NWs Top Electrode

**DOI:** 10.1021/acsami.4c11431

**Published:** 2024-09-26

**Authors:** Daojian Su, Ting Ding, Peili Gao, Hang Liu, Yinman Song, Guoqiang Yuan, Xin He, Fanyuan Meng, Shuangpeng Wang

**Affiliations:** † School of Applied Physics and Materials, Wuyi University, Jiangmen 529020, P. R. China; ‡ Jiangmen Key Laboratory of Micro-Nano Functional Materials and Devices, Jiangmen 529020, P. R. China; § Joint Key Laboratory of the Ministry of Education, Institute of Applied Physics and Materials Engineering, University of Macau, Avenida da Universidade, Taipa, Macau 999078, P. R. China

**Keywords:** transparent electrodes, MXene, silver nanowires, interface engineering, organic light-emitting diodes

## Abstract

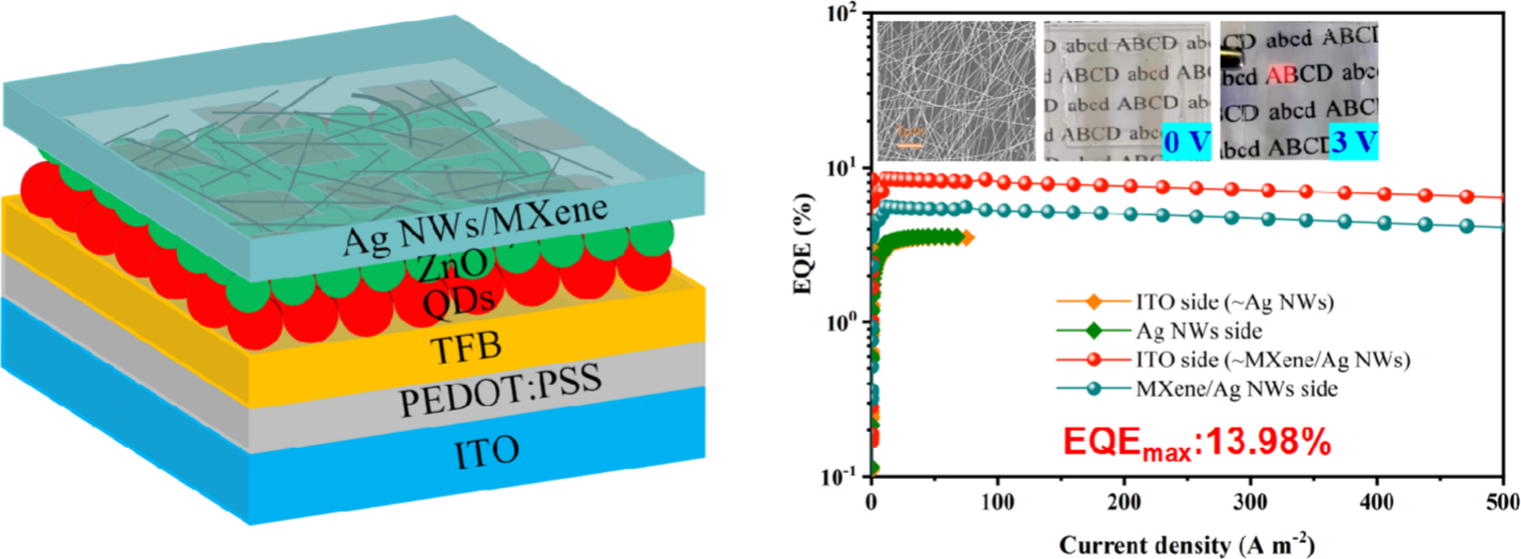

The integration of high-performance transparent top electrodes with the functional layers of transparent quantum dot light-emitting diodes (T-QLEDs) poses a notable challenge. This study presents a composite transparent top electrode composed of MXene and Ag NWs. The composite electrode demonstrates exceptional transparency (84.6% at 620 nm) and low sheet resistance (16.07 Ω sq^–1^), rendering it suitable for integration into T-QLEDs. The inclusion of MXene nanosheets in the composite electrode serves a dual role: adjusting the work function to enhance electron injection efficiency and enhancing the interface between Ag NWs and the emissive layer, thereby mitigating the common issue of interfacial resistance in conventional transparent electrodes. This strategic amalgamation results in notable improvements in device performance, yielding a maximum current efficiency of 23.12 cd A^–1^, an external quantum efficiency of 13.98%, and a brightness of 21,015 cd m^–2^. These performance metrics surpass those achieved by T-LEDs employing pristine Ag NW electrodes. This study offers valuable insights into T-QLED device advancement and provides a promising approach for transparent electrode fabrication in optoelectronic applications.

## Introduction

With the integration of cutting-edge electronic information technology and the emergence of 5G, we have entered a revolutionary period in optoelectronics, driving rapid innovations in display technologies.^1−4^ Inorganic colloidal quantum dots, especially cadmium selenide (CdSe), have garnered significant attention due to their tunable emission spectra, exceptional color fidelity, and expansive color range, which are ideally compatible with solution-based processing techniques.^5−9^ These attributes make QDs a compelling candidate for the advancement of display technologies.^10−15^ Transparent Quantum Dot Light-Emitting Diodes (T-QLEDs) represent a pioneering advancement in display technology, providing a transformative method for creating screens that are exceptionally transparent, offer vibrant color accuracy, and are adaptable to flexible designs. These characteristics are essential for advancing interactive displays, intelligent windows, and augmented reality devices, laying the foundation for a wave of innovative developments in optoelectronic applications.^16−19^

Traditional metal-based electrodes, typically utilizing vapor-deposited metal films like aluminum, silver, and gold, are commonly employed as the top electrode. Despite their conductivity, these electrodes often lack the required transparency, thereby affecting the visual performance of T-QLEDs.^20−25^ On the contrary, transparent conductive oxides, such as indium tin oxide (ITO) and indium-doped zinc oxide (IZO), offer superior transparency but may not match the conductivity of metals and can be constrained by fabrication complexities and material brittleness.^26−30^ Moreover, the high-temperature processing often required for these materials is incompatible with the organic layers in T-QLEDs, posing a significant challenge for the fabrication of flexible and transparent displays. Therefore, it is imperative to seek an ideal transparent electrode material that effectively integrates these characteristics while maintaining the structural robustness of the device.^8,31−35^

To address these challenges, the emerging two-dimensional layered material Ti_3_C_2_T_X_ (MXene) shows promise due to its abundant unsaturated surface functional groups, exceptional conductivity, and transparency.^36^ Especially, MXene’s substantial specific surface area and profusion of functional groups provide ample adsorption sites, creating favorable conditions for electron transfer and carrier injection.^37^ Research has demonstrated that in solar cells, the adsorption of zinc oxide (ZnO) onto MXene nanosheets forms charge transfer channels.^38^ In perovskite light-emitting diodes, MXene can modulate the work function of ZnO.^39^ Recent studies have shown the use of MXene to regulate the work function of ZnMgO, resulting in a maximum external quantum efficiency (EQE) of up to 15.81% and high-performance blue light QLED.^40^ However, the challenge of balancing the large-area conductivity and transparency of MXene restricts its application as the top electrode in all solution nonsputtering T-QLED. Fortunately, MXene conductive films support low-temperature solution processing and can be combined with Ag NWs to form high-performance transparent electrodes, leveraging the advantages of both materials. In the realm of flexible solar cells, composite electrodes of Ag NWs and MXene have demonstrated applicability in high-performance and highly stable perovskite solar cells and QLED devices.^41,42^

In this study, we examined a composite transparent top electrode consisting of MXene and Ag NWs. The electrode features a MXene transition layer positioned between the ZnO electron transport layer and the Ag NWs conductive network. Notably, the composite electrode exhibits a transmittance of 84.6% at 620 nm, an average visible light transmittance of 86.4%, and a low sheet resistance of 16.07 Ω sq^–1^. Furthermore, we integrated this composite top electrode into red T-QLED devices, following a structure of ITO/PEDOT:PSS/TFB/QDs/ZnO/MXene/Ag NWs. The study demonstrates that the device attains a peak current efficiency (CE) of 23.12 cd A^–1^, a peak EQE of 13.98%, and a maximum brightness of 21,015 cd m^–2^. A comparative evaluation with Ag NWs top electrodes highlights the enhanced performance of MXene/Ag NWs-based transparent electrodes in T-QLED devices. The incorporation of MXene nanosheets decreases the interface resistance between Ag NWs by promoting contact welding, thereby establishing additional charge transfer pathways and facilitating charge transfer between the ZnO layer and Ag NWs electrode. Moreover, the substantial specific surface area and abundant adsorption sites of MXene enable the conversion of point contact between the ZnO layer and Ag NWs electrode into surface contact, leading to improved carrier injection efficiency and overall device performance. This research is anticipated to offer novel insights to advance the development of T-QLED devices.

## Results and Discussion

**Figure 1:**
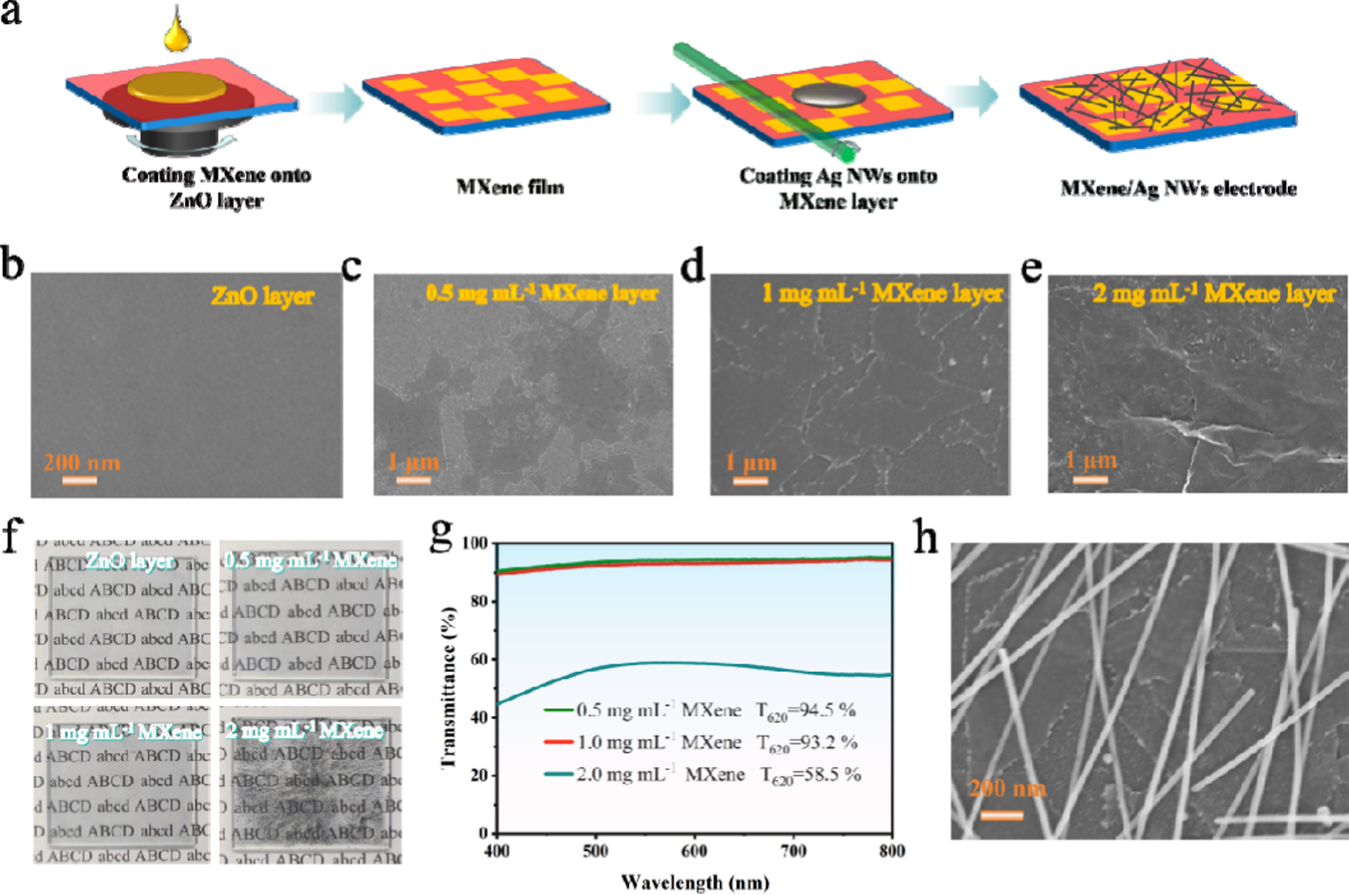
(a) Schematic diagram of preparation process of the MXene/Ag NWs composite electrode; SEM images of ZnO layer (b), 0.5 mg mL^–1^, 1.0 mg mL^–1^ and 2.0 mg mL^–1^ MXene layers (c–e); Corresponding physical photos (f), and (g) Transmittance spectra of the thin films based on MXene at different concentrations; (h) SEM image of the MXene/Ag NWs coated on the ZnO layer.

The process of fabricating the MXene/Ag NWs composite transparent electrode is depicted in Figure 1a. The electrode comprises a double-layer composite top electrode, where the MXene layer and Ag NW conductive network layer are successively deposited onto the ZnO electron transport layer. The quality of the MXene layer significantly affects the device’s performance. Challenges such as nonuniform film distribution caused by the coffee ring effect during the spin coating of MXene nanosheets, and excessive coverage leading to decreased transmittance, can adversely impact the stability and optical properties of the device. Hence, it is crucial to control the MXene concentration to fulfill the device’s performance criteria.

The study examined the influence of MXene concentration on the performance of transparent top electrodes through the utilization of a spin-coating technique to fabricate MXene films at concentrations of 0.5, 1.0, and 2.0 mg mL^–1^ on ZnO layers. Initially, the pure ZnO layer exhibited a compact, even surface composed of small nanoparticles (Figure 1b). With increasing MXene concentration, the deposition of MXene on the ZnO layer significantly enhanced. At 0.5 mg mL^–1^, the coverage of the MXene film was relatively sparse, with nanosheets dispersed throughout (Figure 1c). However, at 1.0 mg mL^–1^, the MXene coverage substantially increased, resulting in the formation of a continuous, conductive thin layer (Figure 1d).

Further increasing the MXene concentration to 2.0 mg mL^–1^ resulted in the complete envelopment of the ZnO layer by the MXene layer, with a distinct folded structure primarily due to the stacking of multiple MXene layers (Figure 1e). The corresponding physical photos indicate that the introduction of a low concentration of MXene did not significantly reduce the transparency of the ZnO layer, allowing the text beneath the film to remain distinguishable (Figure 1f). As the concentration of MXene increased to 2.0 mg mL^–1^, pronounced coffee ring effects emerged, leading to unevenly dispersed black spots and a significant decrease in film transparency. Figure 1g depicts the transmittance curves of MXene films at varying concentrations. At MXene concentrations of 0.5 and 1.0 mg mL^–1^, the transmittance at 620 nm was 94.5 and 93.2%, respectively, resulting in an average transmittance of 93.9 and 92.8% within the visible light range. However, at a concentration of 2.0 mg mL^–1^, the film’s transmittance notably decreased to 58.5% at 620 nm, with an average transmittance of 55.5%. Additionally, optical microscopy images revealed the formation of black aggregates at a high MXene concentration, leading to a significant reduction in electrode transparency. This limitation hinders its potential as a transparent top electrode in T-QLED (Figure S1).

The square resistance plays a crucial role in determining the performance of electrodes. A lower concentration of MXene layers leads to a dispersed distribution of MXene nanosheets onto the ZnO layer, resulting in the formation of disconnected conductive networks and higher electrical resistivity. Therefore, we measured the average square resistance of MXene layers at a concentration of 2.0 mg mL^–1^, which was found to be 366.2 Ω sq^–1^, indicating a low conductivity that could potentially affect charge carrier injection. Achieving a balance between the optical transmittance and conductivity of pure MXene electrodes is challenging. To address this issue, we applied a coating of Ag NWs conductive networks onto the MXene layer to optimize the optoelectronic performance of the top electrode. The contact angle of Ag NWs on pure ZnO and MXene films prepared with concentrations of 0.5, 1.0, and 2.0 mg mL^–1^ were 12, 11.4, 10.2, and 7°, respectively (Figure S2), indicating that higher MXene concentrations enhance wettability by increasing the adsorption sites, thereby facilitating further coating of the Ag NWs layer. The average square resistances of MXene/Ag NWs composite transparent electrodes were 20.01, 16.07, and 12.82 Ω sq^–1^, demonstrating a significant improvement in conductivity compared to pristine MXene films. The composite electrode exhibits photoelectric properties comparable to commercial ITO electrodes, establishing it as an excellent material for transparent top electrodes. Figure S3 presents the low-magnification SEM images of these electrodes, which effectively showcase the distinct and cross-aligned network structure of the Ag NWs in both the pure Ag NW and MXene/Ag NW composite electrodes. The SEM images demonstrate that the incorporation of the MXene layer does not notably change the organization of the Ag NW network, preserving the desired conductivity and structural stability of the electrode. The high-magnification SEM image of the MXene/Ag NWs composite transparent electrode illustrates the role of MXene nanosheets as a carrier transport bridge between ZnO nanoparticles and Ag NWs (Figure 1h). The MXene nanosheets increase the contact area between ZnO and Ag NWs, while those positioned at the nodes of the nanowires effectively reduce interface contact resistance. This enhancement boosts the electron transfer capacity within the conductive network of the top electrode.

**Figure 2:**
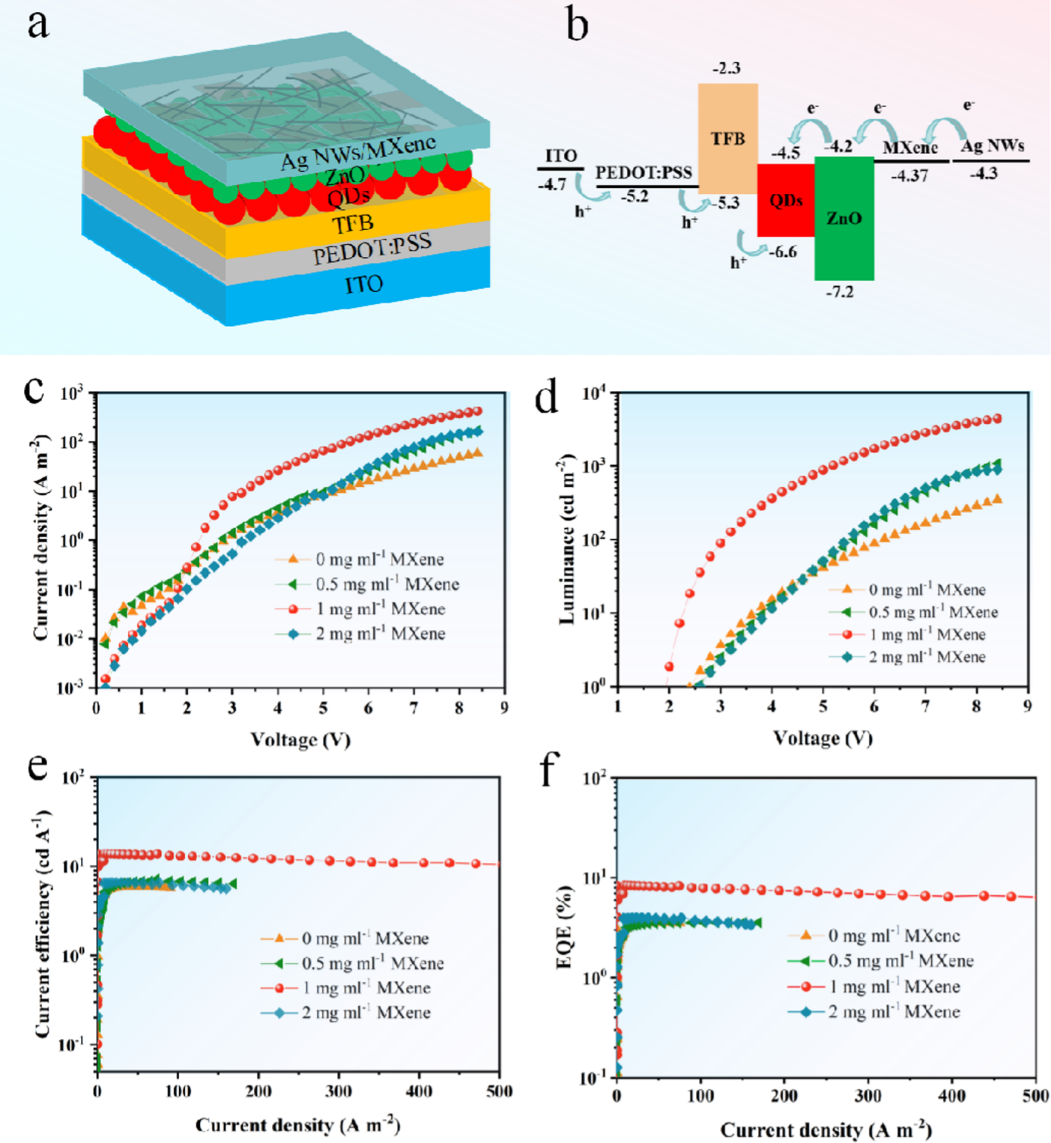
(a) Schematic structure of the T-QLED device; (b) Energy level diagram of the functional layers within the device; (c) Current density–voltage, (d) Luminance–voltage; (e) Current efficiency-current density; (f) External quantum efficiency-current density curves of the T-QLED devices based on MXene at different concentrations.

To assess the viability of utilizing the MXene/Ag NWs composite electrode in T-QLEDs, we designed a device with the following structure: ITO/PEDOT:PSS/TFB/QDs/ZnO/MXene/Ag NWs, as illustrated in Figure 2a. In this configuration, ITO functions as the bottom electrode, PEDOT:PSS serves as the hole injection layer, and TFB acts as the hole transport layer (HTL). The emissive layer (EML) of the device consists of red-emitting CdSe/ZnS quantum dots, while ZnO is employed as the electron transport layer (ETL), and the top electrode is composed of MXene/Ag NWs. The microstructure and optical properties of CdSe/ZnS quantum dots are presented in Figures S4 and S5. The red quantum dots exhibit a peak emission at 620 nm, with a fluorescence quantum efficiency of 72.2%. Furthermore, the prepared ZnO nanoparticles exhibit excellent dispersibility, with an average particle size of approximately 5.9 nm (Figure S6), facilitating effective interface contact with the top electrode and the quantum dot emissive layer, thereby promoting electron transport. The energy level constraints of the device are illustrated in Figure 2b, with the MXene layer at −4.37 eV and the Ag NWs layer at −4.3 eV (Figure S7 and eq S1).^43^ The closely matched energy levels in the composite electrode structure signify an alignment in energy levels, crucial for minimizing electron energy loss between the electrode and the functional layer, reducing interface defects, and enhancing electron injection.

The impact of different concentrations of MXene layers (0.5, 1.0, and 2.0 mg mL^–1^) on the performance of T-QLED devices was investigated. Figure 2c illustrates the current density–voltage curve of the device, indicating a significant leakage current at an MXene concentration of 0.5 mg mL^–1^. This can be attributed to incomplete coverage of the low-concentration MXene layer, leading to charge imbalance. Increasing the MXene concentration to 1.0 mg mL^–1^ notably reduces the device leakage current and enhances current density by improving MXene layer coverage, facilitating uniform charge injection, and enhancing electron transfer efficiency. However, a further increase in MXene concentration to 2.0 mg mL^–1^ results in decreased current density, likely due to excessive MXene causing uneven layer thickness and local stacking, leading to irregular electron injection and increased electron transport pathways. Subsequent performance tests demonstrated that the device achieved optimal performance at 1 mg mL^–1^ of MXene. At this concentration, the illumination voltage decreased to 1.9 V, and the device reached a maximum brightness of 4257 cd m^–2^ (Figure 2d). The analysis of the current efficiency-current density curve (Figure 2e) and the external quantum efficiency-current density curve (Figure 2f) indicated that the device achieved a peak CE of 13.92 cd A^–1^ and a peak EQE of 8.42%. Consequently, for subsequent investigations, MXene at a concentration of 1 mg mL^–1^ was identified as the optimal transition layer between the ZnO layer and the Ag NWs electrode. This configuration resulted in a transmittance of 84.6% at 620 nm for the MXene/Ag NWs transparent top electrode, with transmittance across the entire visible light spectrum comparable to that of commercial ITO electrodes (Figure S8).

**Figure 3:**
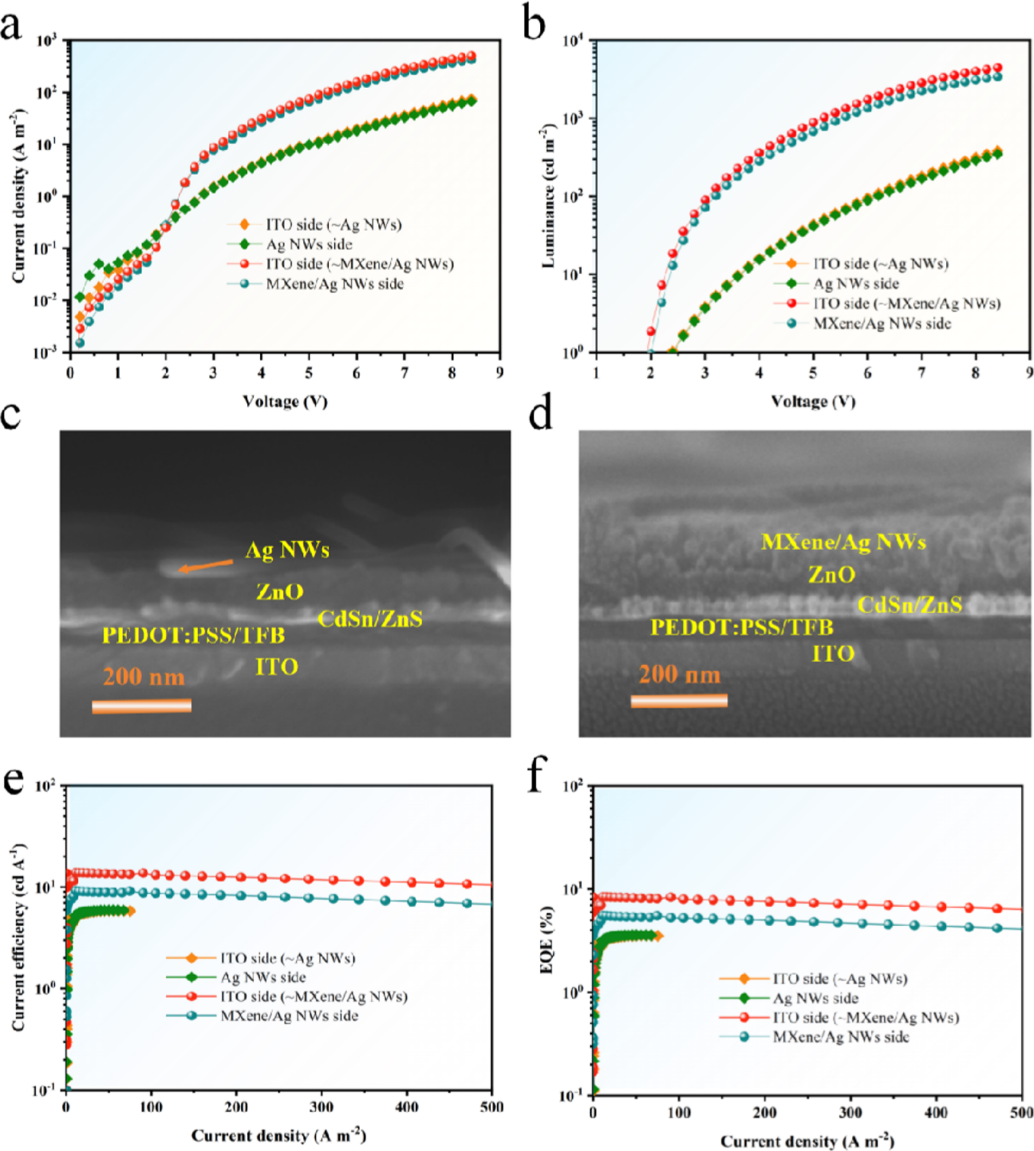
Performance comparison of the T-QLED devices based on the MXene/Ag NWs and Ag NWs top electrodes. (a) Current density–voltage; (b) Luminance–voltage; (e) Current efficiency-current density; (f) External quantum efficiency-current density curves; The cross-section SEM images of T-QLED with an Ag NWs electrode (c), and T-QLED with a MXene/Ag NWs electrode (d).

An evaluation was conducted to assess the effectiveness of MXene/Ag NWs composite top electrodes in T-QLED devices, in contrast to a transparent device featuring an ITO/PEDOT:PSS/TFB/QDs/ZnO/Ag NWs configuration. The current density–voltage curve (*J–V*) illustrates successful suppression of leakage current in the MXene/Ag NWs composite electrode-based device, leading to a significantly higher current density compared to the device utilizing a single-layer Ag NWs electrode (Figure 3a). Additionally, the brightness-voltage curve (*L*-*V*) demonstrates that the total brightness of the device utilizing the MXene/Ag NWs composite electrode reached 21,015 cd m^–2^. Specifically, the brightness on the ITO side was 12,007 cd m^–2^, while on the MXene/Ag NWs side, it was 9008 cd m^–2^, surpassing the total brightness of the Ag NWs-based device at 5016 cd m^–2^ (with 2507 cd m^–2^ on the ITO side and 2509 cd m^–2^ on the Ag NWs side) as shown in Figure 3b. The limited charge transfer channels resulting from the point-to-point contact between 30 nm diameter Ag NWs and 5.9 nm average-sized ZnO nanoparticles hinder charge carrier injection, as depicted in Figures S9a and 3c. In contrast, MXene films, characterized by a large specific surface area and abundant surface adsorption sites, enhance the contact area with ZnO and Ag NWs layers, as illustrated in Figures S9b–d, and 3d. Consequently, an optimal concentration of the MXene layer promotes an increase in charge transfer channels, leading to a rapid augmentation in current density and device brightness, along with a reduction in the turn-on voltage (*V*_on_) from 2.4 to 1.9 V.

The XRD pattern of the MXene/Ag NWs composite electrode reveals distinct diffraction peaks attributed to Ag NWs and MXene, with no new crystal diffraction peaks observed (Figure S10). This observation suggests a physical interaction between the MXene layer and the Ag NWs layer. Additionally, the Zeta potentials of the MXene and Ag NWs dispersion showed a prominent peak at −75 mV, indicating outstanding dispersibility of these materials and facilitating the formation of a homogeneous electrode layer. However, upon mixing the two conductive materials, this peak disappeared. The Zeta potential increased from −4.45 mV for MXene and −8.39 mV for Ag NWs to −3.59 mV for the MXene/Ag NWs mixture, suggesting the formation of electrostatic binding between MXene and Ag NWs (Figure S11). This indicates a favorable charge transfer characteristic between MXene and Ag NWs.

The capacitance–voltage characteristics of T-QLED devices utilizing MXene/Ag NWs and Ag NWs electrodes were compared at 10 kHz, as illustrated in Figure S12. The findings suggest that the device employing the MXene/Ag NWs composite electrode exhibits higher capacitance, primarily due to the incorporation of the MXene layer.^44,45^ The capacitance of the device utilizing MXene/Ag NWs and Ag NWs electrode peaks at 1.75 and 1.85 V, respectively, before gradually decreasing.^46−48^ The elevated transition voltage observed in the Ag NWs device serves as evidence of significant charge accumulation resulting from inefficient charge transport,^49^ likely attributable to the point contact of Ag NWs with ZnO. The presence of a MXene layer enhances the specific surface area of the electrode, thereby facilitating additional pathways for charge transport. The increased capacitance indicates an improved charge storage capacity of the electrode, consequently enhancing charge injection efficiency. A comparative analysis of the current–voltage characteristics of two device configurations, namely ITO/ZnO/MXene/Ag NWs and ITO/ZnO/Ag NWs, is presented in Figure S13.^50,51^ The device incorporating the transparent top electrode of MXene/Ag NWs demonstrates higher current values, aligning with the trends depicted in Figure 3a.

The T-QLED device utilizing a MXene/Ag NWs composite electrode exhibited a maximum CE of 23.12 cd A^–1^ (13.92 cd A^–1^ on the ITO side and 9.20 cd A^–1^ on the MXene/Ag NWs side) and a maximum EQE of 13.98% (8.42% on the ITO side and 5.56% on the MXene/Ag NWs side), as shown in Figures 3e,f, and Table 1. These values surpass significantly the maximum CE of the Ag NWs-based T-QLED device, which was 11.09 cd A^–1^ (5.86 cd A^–1^ on the ITO side and 5.23 cd A^–1^ on the Ag NWs side), as well as a maximum EQE of 6.68% (3.53% on the ITO side and 3.15% on the Ag NWs side). Figure S14 illustrated the peak EQE values from 20 individual devices employing both the MXene/Ag NWs and Ag NWs top electrode configurations. The histogram demonstrates an average EQE of 7.25% for devices with the MXene/Ag NWs composite top electrode and 2.85% for those utilizing the Ag NWs-only top electrode. These statistical results underscore the excellent repeatability of our devices. Furthermore, at a current density of 500 A m^–2^, the T-QLED device utilizing the MXene/Ag NWs composite electrode retained 75% of its original device efficiency, while the Ag NWs-based T-QLED device only operated at a maximum current density of 70 A m^–2^. Additionally, we compared the T-QLEDs with MXene/Ag NWs as transparent top electrodes in our study to those reported in the literature (Table S1), highlighting the significant performance enhancement of T-QLED devices with the incorporation of MXene/Ag NWs as the transparent top electrode.

**Table 1:** Comparison of Performance parameters of MXene/Ag NWs based- and Ag NWs based-T-QLED Devices

top electrode	electrode side	*V*_on_ (V)	*L*_max_ (cd m^–2^)	EL (nm)	CE_max_ (cd A^–1^)	EQE_max_ (%)
MXene/Ag NWs	ITO side	1.9	12,007	620	13.92	8.42
	MXene/Ag NWs side	2.0	9008	620	9.20	5.56
	All	1.9	21,015	620	23.12	13.98
Ag NWs	ITO side	2.4	2507	620	5.86	3.53
	Ag NWs side	2.4	2509	620	5.23	3.15
	All	2.4	5016	620	11.09	6.68

**Figure 4:**
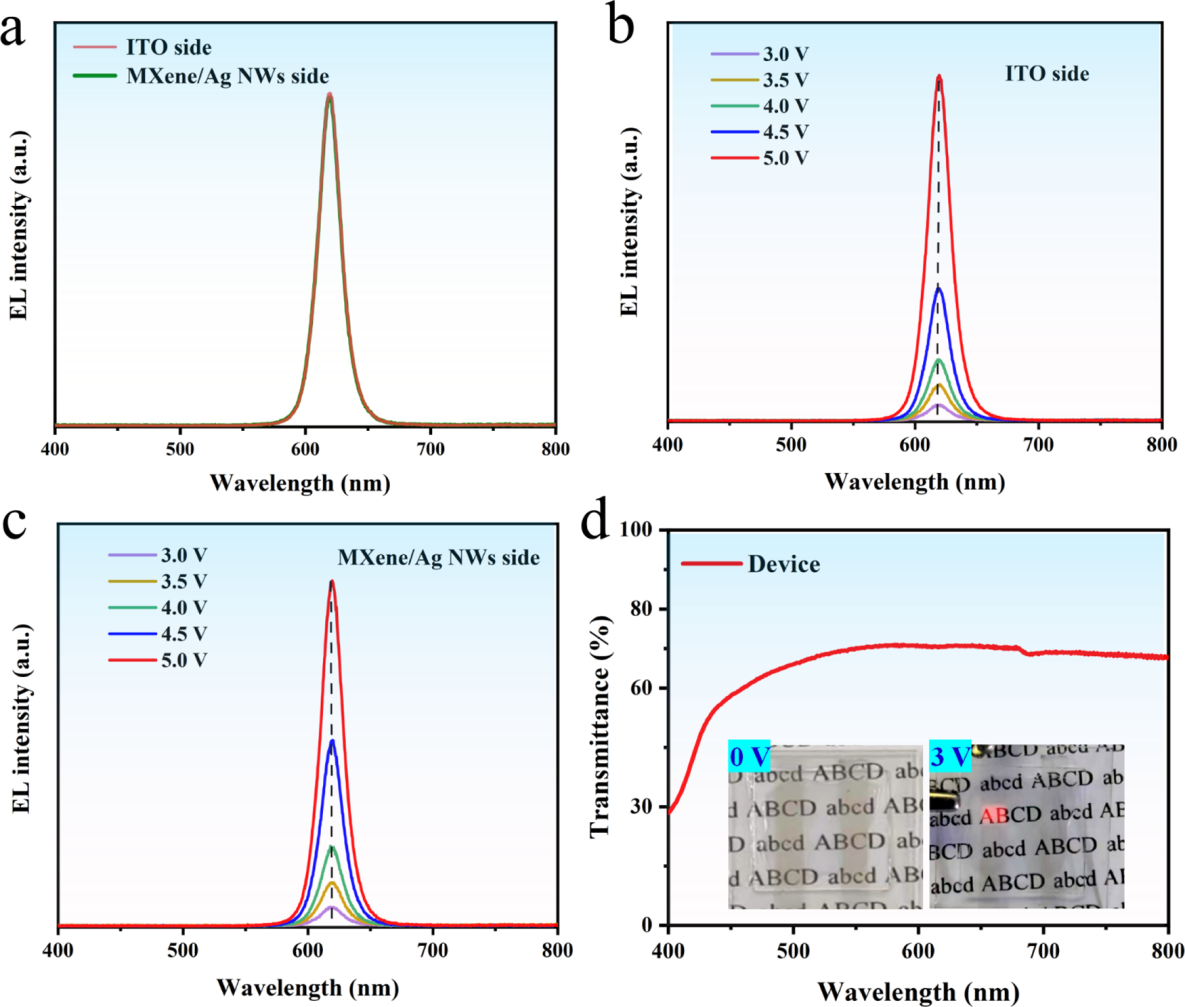
(a) Electroluminescence (EL) spectra of the MXene/Ag NWs-based T-QLED device from both electrode sides; Electroluminescence spectra from the bottom electrode ITO side (b) and top electrode MXene/Ag NWs side at different voltages (c); (d) Transmittance curve of the MXene/Ag NWs-based T-QLED.

The spectral stability of transparent devices is crucial for their operational efficiency. As shown in Figure 4a, we examined the electroluminescence (EL) spectra of the T-QLED based on MXene/Ag NWs composite electrode. The EL peak at 620 nm with a half peak width of 21.7 nm, indicating high color purity. The complete spectral overlap of the EL spectra from the bottom electrode (ITO side) and the top electrode (MXene/Ag NWs side) underscores consistency in the luminescence mechanisms across both electrodes. Further analysis of the EL spectra from the bottom electrode ITO side (Figure 4b) and the top electrode side (Figure 4c) at different voltages reveals no emission peak shifts, confirming the remarkable spectral stability of the device. The T_50_ lifetime of devices utilizing MXene/Ag NWs and Ag NMs as the transparent top electrode is 1.7 and 0.04 h, respectively. In comparison to devices employing Ag NWs, those incorporating MXene/Ag NWs as the transparent top electrode demonstrate a notably enhanced operational lifetime, as illustrated in Figure S15. This steadfastness is essential for ensuring sustained performance and reliability over extended periods for the T-QLED devices. As shown in Figure S16, we further study the effect of temperature on the device stability. Initially, the EQE shows a positive correlation with heating duration, with this relationship diminishing at higher temperatures, attributed to positive aging effects.^52−54^ Conversely, in the subsequent phase, EQE decreases with prolonged heating, particularly accelerated at elevated temperatures, indicating expedited roll-off. Noteworthy is the device’s robust thermal stability at 40 °C; however, stability diminishes with escalating temperatures. The device demonstrates an average transmittance of 55.5% in the visible light range, with a specific transmittance of 70.5% at 620 nm, as shown in Figure 4d, meeting commercial transparency standards. The insets of Figure 4d depict the device under unlit conditions and at a working voltage of 3 V, revealing excellent transparency in both scenarios, as the letters on the back of the device remain visible. As a result, the T-QLED device developed in this study, utilizing the MXene/Ag NWs composite transparent top electrode, exhibits satisfactory device performance and a wide operating current range, indicating promising applications in electronic devices such as transparent touch screens, optoelectronic devices, smart windows, invisible color-changing sensors, and virtual reality technologies.

We also employed MXene/Ag NWs as the transparent top electrode in green T-QLEDs to showcase the general applicability of our method. The *J–V* curve, depicted in Figure S17a, demonstrates a significant increase in current density in the green device, indicating improved carrier injection with our composite transparent top electrode. Additionally, as illustrated in Figure S17b, the turn-on voltage of the MXene/Ag NWs device decreased from 2.9 to 2.3 V, showcasing a notable enhancement in carrier injection for the green device. The maximum current efficiencies for devices utilizing Ag NWs and MXene/Ag NWs were 4.58 and 9.46 cd A^–1^, respectively (Figure S17c), with corresponding maximum EQE of 0.96% and 1.99% (Figure S17d). These enhanced device performances validate the wide applicability of our approach.

## Conclusions

In conclusion, we have developed a transparent top electrode composite of MXene/Ag NWs, featuring a low square resistance of 16.07 Ω sq^–1^ and high transmittance of 84.6% at 620 nm. The incorporation of an MXene transition layer optimized the interface contact between the Ag NW top electrode and the ZnO functional layer, thereby enhancing the charge transfer channel within the electrode to improve carrier injection efficiency. The red T-QLED constructed using this electrode exhibited promising performance metrics, including a transmittance of 70.5% at 620 nm, a minimum turn-on voltage of 1.9 V, a maximum brightness of 21,015 cd m^–2^, and a maximum CE of 23.12 cd A^–1^. This study introduces a viable approach for enhancing T-QLED performance, offering valuable insights for future research and the advancement of optoelectronic devices.

## Materials and Methods

### Materials

The ITO substrate with a sheet resistance of 45 Ω sq^–1^ was obtained from Wuhu Jinghui Electronic Technology Co., Ltd. Zn(OAc)_2_·2H_2_O (99%) was purchased from Alfa Aesar, China, Ltd. MXenes (Ti_3_C_2_T_X_, ethanol) at a purity of 99.9% were sourced from FoShan XinXi Technology Co., Ltd. Potassium hydroxide (99%) was acquired from Sinopharm Chemical, China. The PEDOT:PSS (AI 4083) was purchased from Heraeus. TFB (Poly[(9,9-dioctylfluorene-2,7-diyl)-*co*-(4,4′-(N-(4-*tert*-butylphenyl) diphenylamine))]) was obtained from Sigma-Aldrich. The red quantum dot luminescent material (CdSe/ZnS) was procured from Guangdong PuJiaFu Optoelectronics Technology Co., Ltd. Ag NWs in ethanol, with an average diameter of 30 nm and a length of 20 μm, were sourced from Xianfeng Nanotechnology Co., Ltd. All chemicals were used as received.

### Preparation of ZnO Nanoparticle Dispersion

Initially, 0.025 mol of Zn(OAc)_2_·2H_2_O (99%, Alfa Aesar, China) underwent vacuum dehydration in the condensation reflux apparatus at 120 °C. Subsequently, 150 mL of ethanol was introduced into the condensation reflux apparatus and agitated at 80 °C for 30 min, resulting in the formation of a transparent precursor. Following this, 2 g of potassium hydroxide were dissolved in 20 mL of ethanol, and the resulting solution was gradually added to the precursor. The mixture was stirred for 5 min, leading to the formation of a white ZnO precipitate. Finally, the washed sample was dispersed in ethanol at a concentration of 50 mg mL^–1^. Ethanolamine (typically 50 μL per 20 mL solution) was introduced for the stabilization of ZnO nanoparticles (NPs), rendering it for further use.

### Fabrication of T-QLED Devices

The T-QLED devices are structured with ITO/PEDOT:PSS/TFB/QDs/ZnO/TEs. The ITO substrate, functioning as the bottom electrode, was cleaned ultrasonically with deionized water and ethanol. A 40 nm thick hole injection layer of PEDOT:PSS was spin-coated onto the UV hydrophilic-treated ITO substrate at 3000 rpm for 40 s, then annealed at 150 °C for 20 min. Subsequent layers were fabricated in a nitrogen-filled glovebox. TFB, with a concentration of 8 mg mL^–1^ in chlorobenzene, was spin-coated onto the PEDOT:PSS layer at 3000 rpm for 40 s, followed by annealing at 130 °C for 10 min. CdSe/ZnS QDs, at a concentration of 20 mg mL^–1^ in *n*-octane, were spin-coated onto the TFB layer at 2000 rpm for 40 s, then thermally treated at 100 °C for 10 min. Next, a ZnO dispersion at a concentration of 50 mg mL^–1^ was spin-coated onto the QDs layer and thermally treated at 100 °C for 10 min. The MXene dispersions are applied onto the ZnO film at concentrations of 0, 0.5, 1.0, and 2.0 mg mL^–1^ in ethanol under an inert nitrogen atmosphere. Subsequently, spin-coating is performed at 3000 rpm for 40 s. Following this, Ag NWs with an average diameter of 30 nm, dispersed in ethanol at a concentration of 1 mg mL^–1^, are uniformly deposited onto the MXene layer using a Mayer bar. This deposition process is repeated 10 times to ensure uniformity. The electrode fabrication is completed by annealing the MXene/Ag NWs layer at 100 °C for 10 min, resulting in the formation of the composite transparent top electrode.

### Characterization

The sheet resistance of the transparent electrode was determined using a four-probe tester (M-6, Suzhou Gingge, China). The contact angle of MXene layers was determined using a contact angle/surface tension measuring instrument (LSA100, Lauda Scientific, Germany). The zeta potentials of Ag NWs, MXene, and MXene/Ag NWs dispersion were characterized using a potential analyzer (ZS90, Silver Zetasizer Nano, England). The crystal phase of ZnO NPs was identified through high-resolution transmission electron microscopy (HRTEM; JEOL JEM-F200, FEI, Japan). The thickness of the functional layers and the surface morphology of the conductive network layer were assessed using a focused ion beam scanning electron microscope (SEM; Carl Zeiss, Germany). The absorption of CdSe/ZnS solution (1 mg mL^–1^) and transmission spectra of the transparent electrode were measured employing a UV–visible spectrophotometer (UV-2600, Shimadzu, Japan). The electroluminescence (EL) and photoluminescence (PL) spectra of the devices were recorded using a fiber optic spectrometer (Maya 2000PRO, Ocean Optics). The work functions of the electrodes were determined by ultraviolet photoelectron spectroscopy (UPS; Thermo Scientific ESCALAB Xi). The CE and EQE were measured (eq S2) and calculated by using a constant current source (Keithley2400) and a luminance meter (LS-160, Konica Minolta, Japan).^51^

## Supporting Information Available

The optical microscopy images of zinc oxide and MXene films; The contact angle of AgNWs with zinc oxide and MXene films; SEM image of the Ag NWs, and MXene/Ag NWs conductive network; TEM images of CdSe/ZnS core–shell QDs and energy dispersive spectroscopy; UV–vis absorption and PL spectra; TEM images and particle size of zinc oxide; UPS spectra of Ti_3_C_2_T*_X_*; Calculation Equation (S1) for WF and Equation (S2) for EQE; Transmittance curves for AgNWs, ITO/glass and MXene/Ag NWs electrodes; SEM images of Ag NWs, MXene and MXene/AgNWs electrodes coated on ZnO film; XRD characteristics of Ag NWs, MXene and MXene/Ag NWs electrode; Zeta potential curves of Ag NWs, MXene and MXene/Ag NWs electrode; Capacitance–voltage characteristic curves of T-QLED devices; Current–voltage curves of ITO/ZnO/MXene/Ag NWs and ITO/ZnO/Ag NWs device; Histogram of peak EQEs; T_50_ lifetime of T-QLEDs with MXene/Ag NWs and Ag NWs transparent electrodes; Thermal stability measures of the Red T-QLEDs at different temperatures; Performance curves of the green T-QLED devices; Comparison of Performance parameters of QLED with transparent top electrode (Table S1) (PDF)
