# Supplementary material for: High-Performance Red Transparent Quantum Dot Light-Emitting Diodes via Fully Solution-Processed MXene/Ag NWs Top Electrode
Source: ACS Appl Mater Interfaces. 2024 Sep 26;16(40):54190–9. doi: 10.1021/acsami.4c11431 (PMC11472265; doi:10.1021/acsami.4c11431)
Supplement: Supplementary file 1 — am4c11431_si_001.pdf [file am4c11431_si_001.pdf]

Supporting Information

High-Performance Red Transparent Quantum Dot Light-Emitting Diodes via Fully Solution-Processed Transparent Top Electrodes

Daojian Su[1,3], Ting Ding[3], Peili Gao[3], Hang Liu[3], Yinman Song[3], Guoqiang Yuan[1,2], Xin He[1,2]*, Fanyuan Meng[1,2]*, Shuangpeng Wang[3]*

[1] School of Applied Physics and Materials, Wuyi University, Jiangmen 529020, P.R. China;

[2] Jiangmen Key Laboratory of Micro-Nano Functional Materials and Devices, Jiangmen, 529020, P.R. China;

[3] Joint Key Laboratory of the Ministry of Education, Institute of Applied Physics and Materials Engineering, University of Macau, Avenida da Universidade, Taipa, Macau 999078, P.R. China.

Correspondances: hexin@wyu.edu.cn (Xin He); mfybys@163.com (Fanyuan Meng); spwang@um.edu.mo (Shuangpeng Wang)

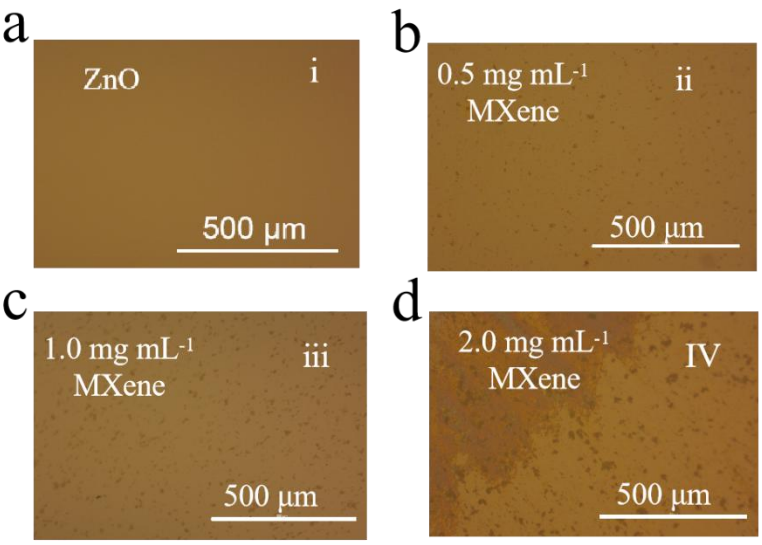

Figure S1. (a) The optical microscopy images of zinc oxide films. The optical microscopy images of MXene films prepared at concentrations of 0.5 mg mL^{-1}, 1.0 mg mL^{-1} and 2.0 mg mL^{-1} (b-d).

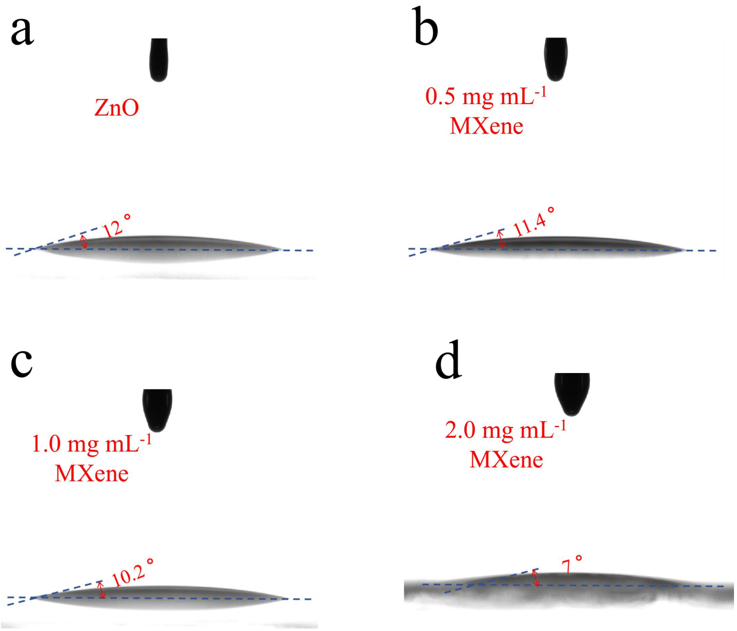

Figure S2. (a) The contact angle of silver nanowires dispersed in an ethanol solution with ZnO films was determined. The contact angle of Ag NWs dispersed in ethanol solution with films prepared from MXene dispersions with concentrations of 0.5 mg mL^{-1}, 1.0 mg mL^{-1} and 2.0 mg mL^{-1} was investigated (b-d).

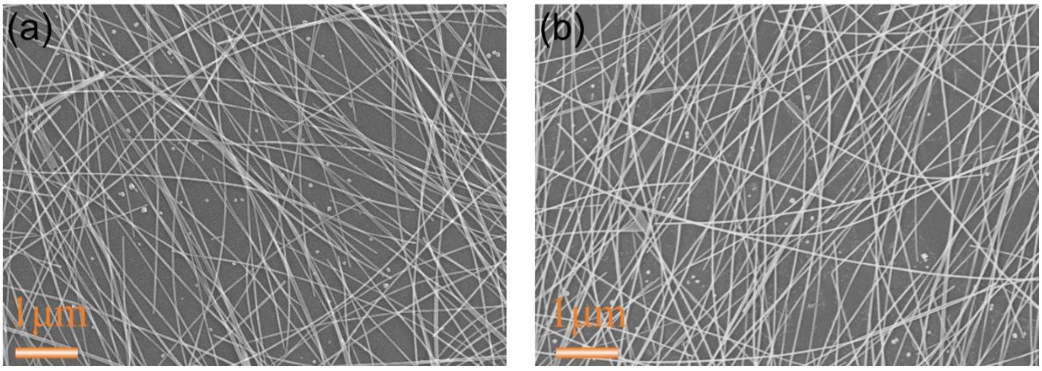

Figure S3. SEM image of the (a) Ag NWs, and (b) MXene/Ag NWs coated on the ZnO layer.

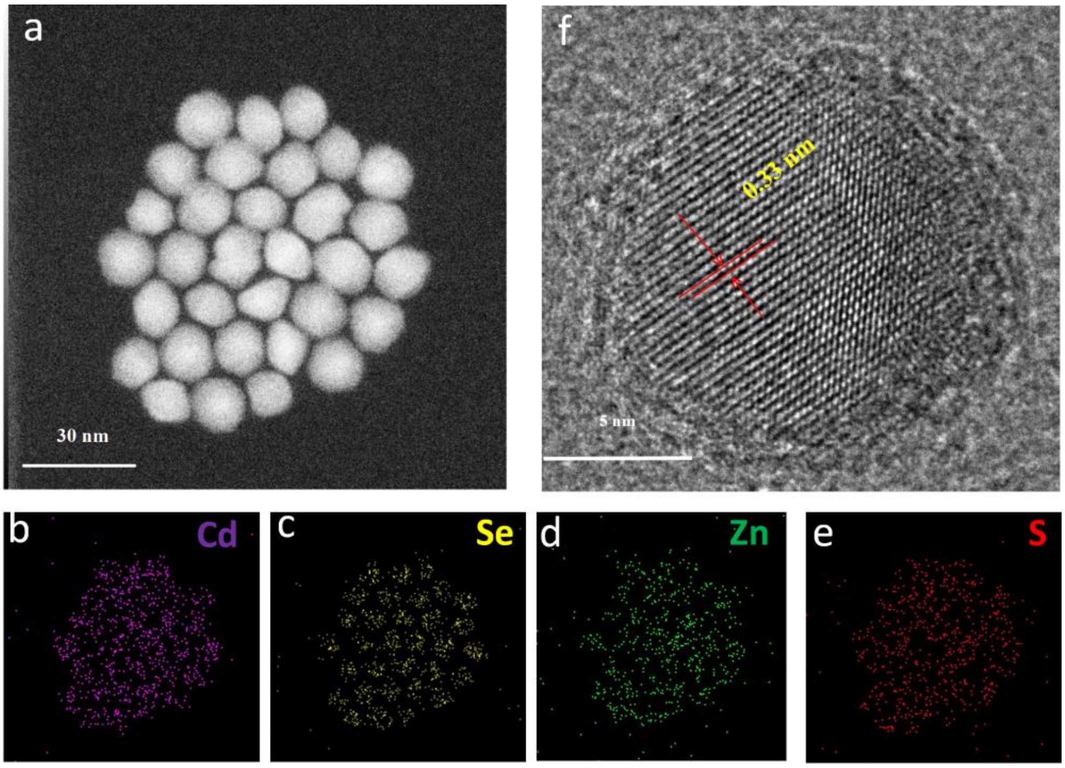

Figure S4. (a) TEM image, (b-e) Energy Dispersive Spectroscopy, and f) HRTEM image of the CdSe/ZnS core-shell QDs.

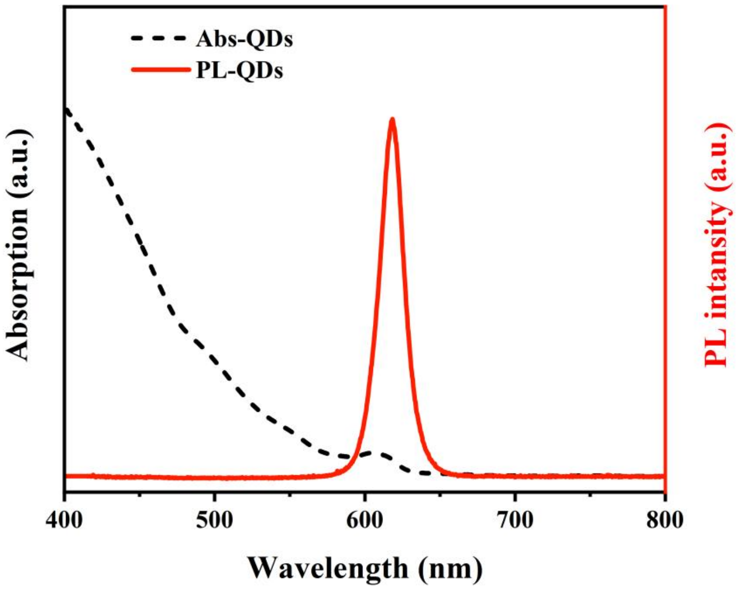

Figure S5. UV-vis absorption and photoluminescence (PL) spectra of QDs.

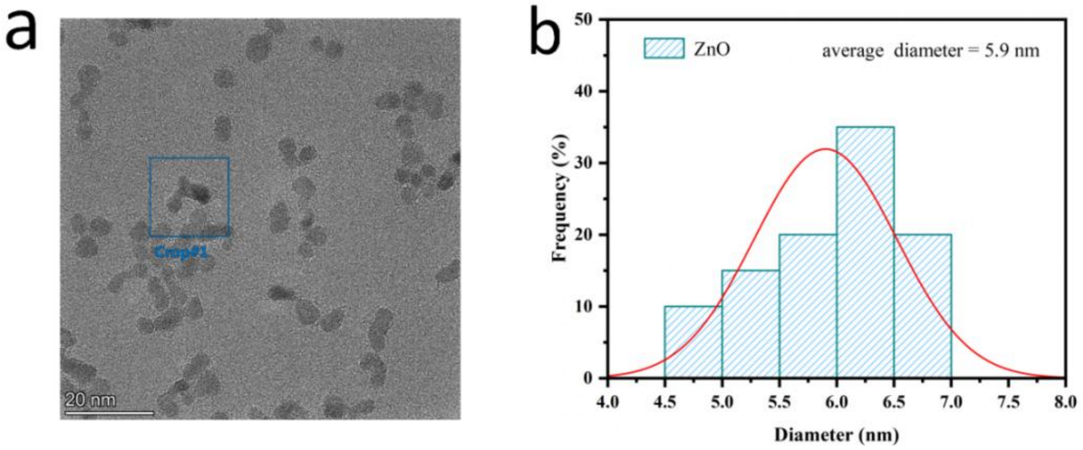

Figure S6. (a) ZnO TEM image. (b) The Diameter of the ZnO nanoparticles.

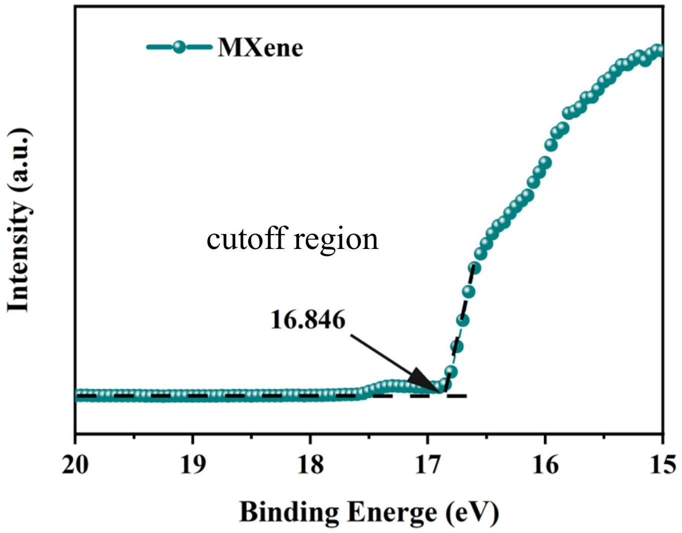

Figure S7. The secondary electron cut-off region UPS spectra of $Ti_3C_2T_X$.

The WF were calculated using the following Equation [(1), (2)]:

$$WF = E_{cutoff} - He\ I(21.22eV) \quad \text{(S1)}$$

The EQE were calculated using the following Equation [(3)]:

$$EQE = \frac{1.18 \times current\ efficiency \times \pi \times Emission\ wavelength}{Visual\ constant \times 10000} \quad \text{(S2)}$$

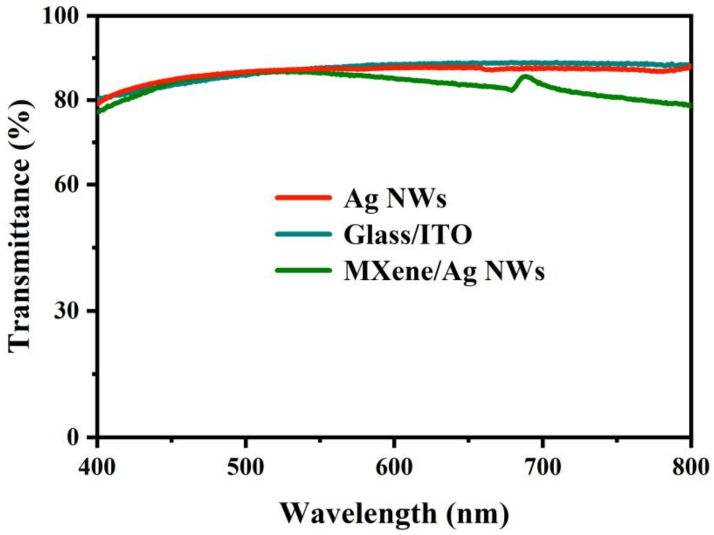

Figure S8. Transmittance curves for Ag NWs, ITO/glass and MXene/Ag NWs electrodes.

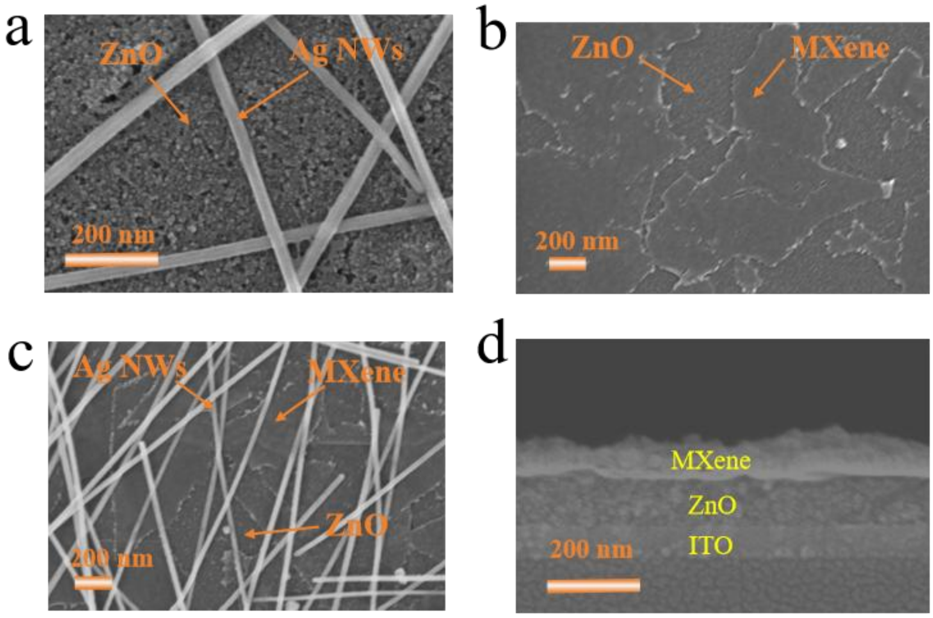

Figure S9. The SEM images of (a) Ag NWs electrodes coated on ZnO film, (b) MXene electrode coated on ZnO film, and (c) composite electrodes of MXene/Ag NWs coated on ZnO film. The cross-section SEM images of (d) MXene electrode coated on ZnO film.

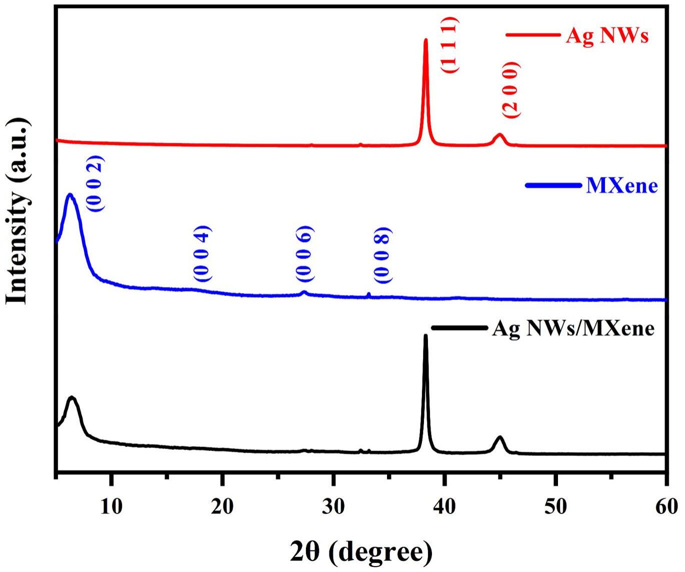

Figure S10. The XRD characteristics of Ag NWs, MXene and MXene/Ag NWs electrode were compared.

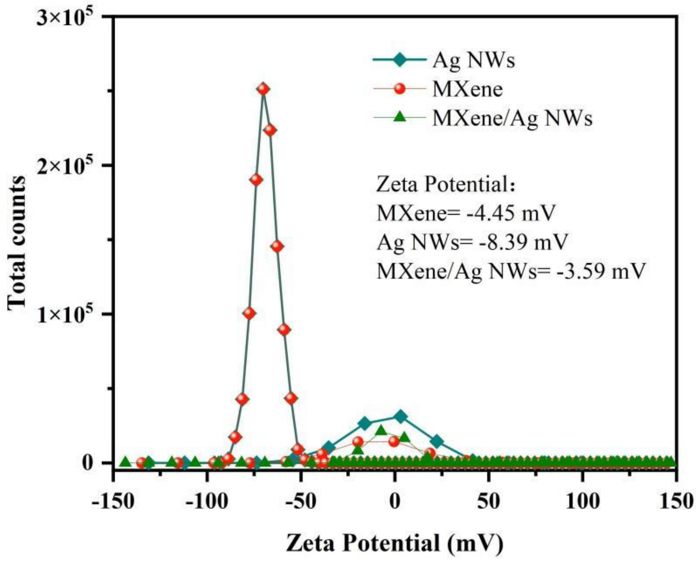

Figure S11. The characteristic zeta potential curves of Ag NWs, MXene and MXene/Ag NWs electrode arewere presented.

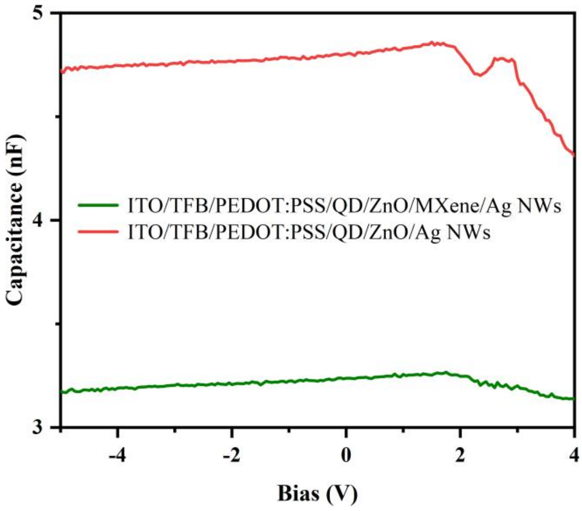

Figure S12. The capacitance-voltage characteristic curves of T-QLED devices prepared from Ag NWs with MXene/Ag NWs at 10 kHz conditions are presented.

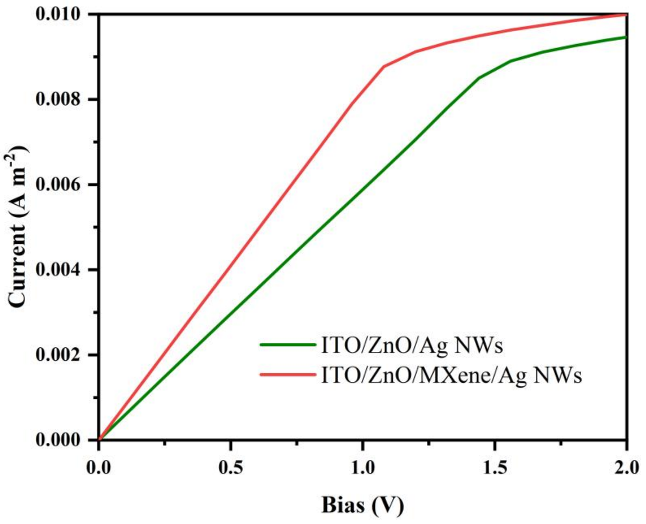

Figure S13. Current-voltage curves of two device structures, ITO/ZnO/MXene/Ag NWs and ITO/ZnO/Ag NWs.

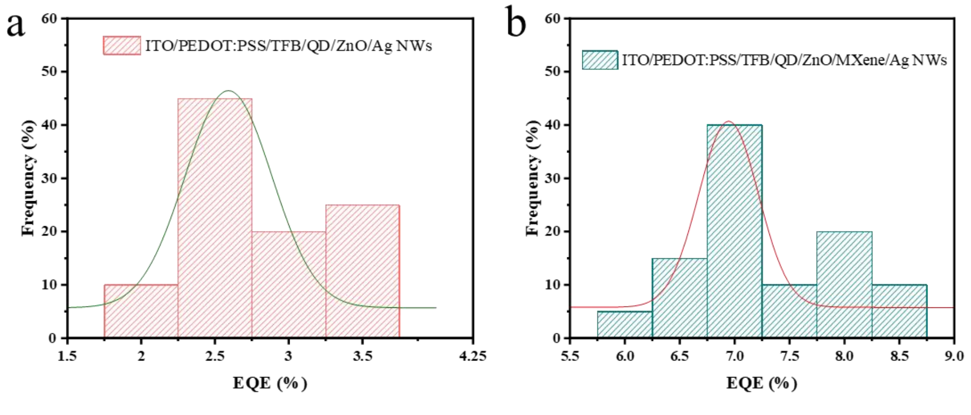

Figure S14. Histogram of peak EQEs of red T-QLED based on (a) Ag NWs (the average EQE was 2.85%) and (b) MXene/Ag NWs (the average EQE was 7.25%) top electrodes with 20 devices.

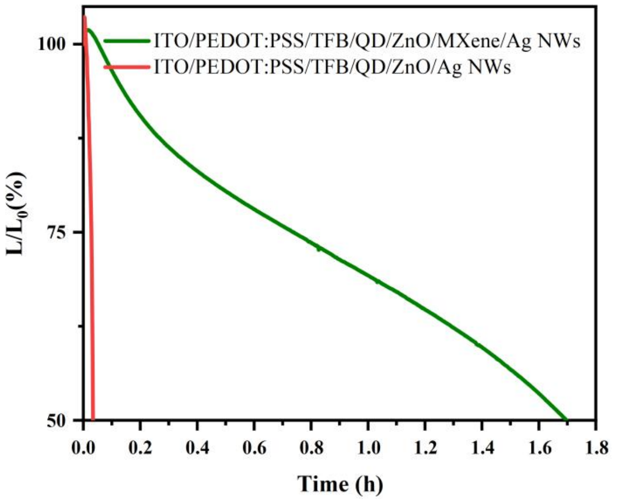

Figure S15. The T_{50} lifetime of the ITO/PEDOT:PSS/TFB/QDs/ZnO/MXene/Ag NWs and ITO/PEDOT:PSS/TFB/QDs/ZnO /Ag NWs devices with an initial luminance of 100 cd/m^2.

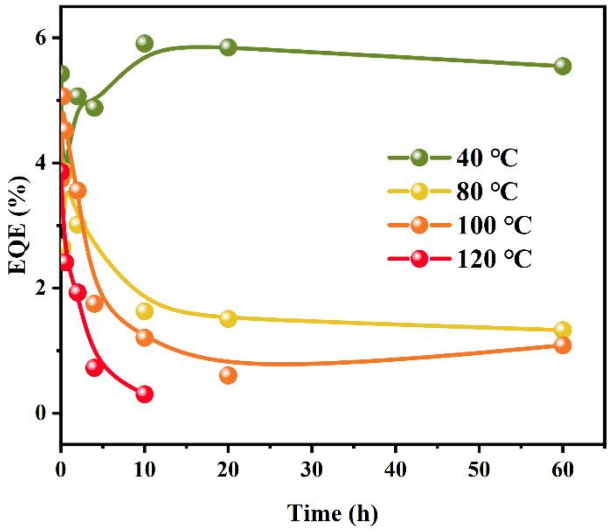

Figure S16. Thermal stability measures of the Red T-QLEDs at different temperatures.

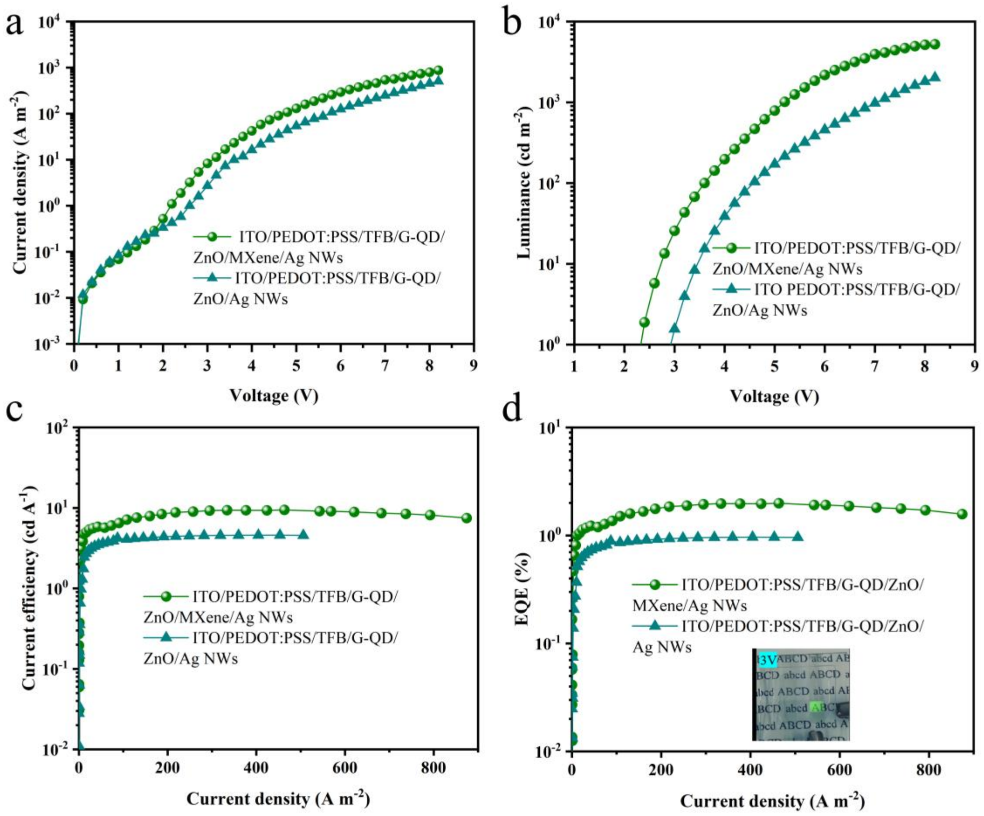

Figure S17. Performance comparison of the green T-QLED devices based on the MXene/Ag NWs and Ag NWs top electrodes. (a) Current density-voltage; (b) Luminance-voltage; (c) Current efficiency-current density; (d) External quantum efficiency-current density curves.

Table S1. Comparison of Performance parameters of T-QLED with transparent top electrode.

S.N	structure	V_{on}	L_{max} (cd m^{-2})	CE_{max}(cd A^{-1})		EQE_{max}(%)		Ref
				ITO side	Ag NWs side	ITO side	Ag NWs side	
1	ITO/PEDOT:PSS/Poly-TPD/ R-QDs/ZnO/Ag NWs	2.5	48,084	5.56	5.20	—	—	(4)
2	ITO/PEDOT:PSS/TFB//R-QD s/ ZnO/Ag NWs	1.8	—	5.43	5.00	5.99	5.44	(5)
3	ITO/PEDOT:PSS/TFB//R-QD s/ ZnO/Ag NWs	3.0	1,778	8.26	9.61	7.17	8.30	(6)
4	ITO/ZnO/G-QDs/PVK/ PEDOT:PSS/Ag NWs	3.0	38,470	10.80	—	6.60	—	(7)
5	ITO/PEDOT:PSS/TFB/R-QD s/ ZnO/MXene/Ag NWs	1.9	21,015	13.92	9.20	8.42	5.56	This work

Reference:

(1) Lu, Q.; Wang, J.; Miao, Y.; Guo, Y.; Wang, G.; Dong, J.; Zhao, M.; Wang, H. UV-ozone Treated $Ti_3C_2T_X$-MXene nanosheets as Hole Injection Layer for Organic Light-emitting Diodes. *Chem. Eng. J.* 2022, 450.138439.

(2) Liang, S.; Wang, S.; Wu, Z.; Wen, B.; Cai, G.; Jiang, X.; Huang, G.; Li, C.; Zhao, Y.; Du, Z. Interfacial Charge Modulation: An Efficient Strategy for Stable Blue Quantum-Dot Light-Emitting Diodes. *Adv. Opt. Mater.* 2022, 11 (2)，202201802.

(3) Dong, J. Y.; Ng, K. W.; Song, Y. M.; Li, J. L.; Kong, Y. C.; Wang, M. W.; Xu, J. C.; Li, L.; Chen, S.; Tang, Z. K.; et al. Observation and Suppression of Stacking Interface States in Sandwich-Structured Quantum Dot Light-Emitting Diodes. *ACS Appl. Mater. Interfaces* 2021, 13 (47), 56630-56637.

(4) Jing, P.; Ji, W.; Zeng, Q.; Li, D.; Qu, S.; Wang, J.; Zhang, D. Vacuum-free Transparent Quantum Dot Light-emitting Diodes with Silver Nanowire Cathode. *Sci. Rep.* 2015, 5 (1), 12499.

(5) Sun, J.; Wang, H.; Shi, H.; Wang, S.; Xu, J.; Ma, J.; Ma, B.; Wen, M.; Li, J.; Zhao, J.; et al. Large-Area Tunable Red/Green/Blue Tri-Stacked Quantum Dot Light-Emitting Diode Using Sandwich-Structured Transparent Silver Nanowires Electrodes. *ACS Appl. Mater. Interfaces* 2020, 12 (43), 48820-48827.

(6) Meng, L.; Zhang, M.; Deng, H.; Xu, B.; Wang, H.; Wang, Y.; Jiang, L.; Liu, H. Direct-Writing Large-Area Cross-Aligned Ag Nanowires Network: Toward High-Performance Transparent Quantum Dot Light-Emitting Diodes. *CCS Chemistry* 2021, 3 (8), 2194-2202.

(7) Zhang, K.; Meng, L.; Zhang, M.; Li, Y.; Jiang, L.; Liu, H. An Ultra-High Transparent Electrode via a Unique Micro-Patterned Ag NWs Crossing-Network with 3.9% Coverage: Toward Highly-Transparent Flexible QLEDs. *Adv. Funct. Mater.* 2023, 34 (4), 202308468.
